# Tackling variants with antibodies

**DOI:** 10.7554/eLife.77751

**Published:** 2022-03-28

**Authors:** Elizabeth H Aitken, Stephen J Rogerson

**Affiliations:** 1 Department of Infectious Diseases, Department of Microbiology and Immunology, at the Doherty Institute, University of Melbourne Melbourne Australia

**Keywords:** pregnancy, malaria, vaccine, VAR2CSA, variant, Placental malaria, blocking antibody, *P. falciparum*

## Abstract

Antibodies targeting the protein that causes placental malaria can recognise multiple variants of the protein, which may help guide the development of new vaccines to protect pregnant women from malaria.

**Related research article** Doritchamou JYA, Renn JP, Jenkins B, Mahamar A, Dicko A, Fried M, Duffy PE. 2022. A single full-length VAR2CSA ectodomain variant purifies broadly neutralizing antibodies against placental malaria isolates. *eLife* **11**:e76264. doi: 10.7554/eLife.76264.

Malaria is a serious, potentially life-threatening disease spread by mosquitoes. Pregnant women are especially at risk, as high densities of the parasite that causes malaria can accumulate in the placenta. This can trigger damaging inflammation in the placenta, which could affect the growth and development of the unborn baby, and even lead to a higher risk of infant death (Walker et al., 2014).

The malaria parasites that infect pregnant women are unique in displaying a protein called VAR2CSA on their surface. When VAR2CSA binds to CSA, a molecule on the surface of placenta cells, it leads to placental malaria. Over successive pregnancies, the body develops immunity against placental malaria by generating antibodies targeting VAR2CSA, and preventing it from attaching to CSA (Salanti et al., 2004). This suggests that VAR2CSA vaccines, which mimic the body’s natural defence mechanisms, may be able to provide some protection against placental malaria.

However, the sequences of the gene that codes for VAR2CSA vary between the different parasite strains (Benavente et al., 2018). Two trial vaccines have so far been developed based on sub-parts of the protein, using different variants of VAR2CSA that included an important CSA-binding domain. And although each vaccine candidate worked well against the variant of the protein used, there was little evidence of protection against other variants (Mordmüller et al., 2019; Sirima et al., 2020). Now, in eLife, Patrick Duffy and colleagues at the US National Institute of Allergy and Infectious Diseases – including Justin Doritchamou as first author – investigated whether an antibody could recognise different versions of the full-length VAR2CSA protein from different parasites (Doritchamou et al., 2022).

Doritchamou et al. first mixed five variants of the VAR2CSA protein (one at a time) with a pooled plasma sample from women who were immune to placental malaria. Each time they mixed a single VAR2CSA variant with the plasma, antibodies that bound to that variant were purified out of the pool and quantified. This revealed that most antibodies had bound to the first two variants, suggesting that antibodies towards the latter variants had already been depleted from the pool because they also recognised the earlier variants.

To confirm that antibodies were cross-protective, the researchers then took the antibodies they had purified using one particular VAR2CSA variant and tested their ability to recognise other variants. This revealed that the antibodies that attached to the first VAR2CSA variant were able to recognise all other tested variants. Cross-recognition was seen using purified proteins or using infected red blood cells expressing the variant.

The fact that naturally acquired antibodies against one version of full-length VAR2CSA also react with other variants implies that exposure to a small number of VAR2CSA variants might be enough to provide protection (Figure 1). This finding differs from the group’s previous results using just domains of VAR2CSA, where antibodies for a specific domain from one variant did not bind to other variants (Doritchamou et al., 2016). This suggests that the important antibody target on the protein may only form when the whole protein folds together. However, VAR2CSA is a large molecule and synthesising the amount of complete protein needed for a vaccine would be challenging, especially if multiple variants are required.

**Figure 1.**
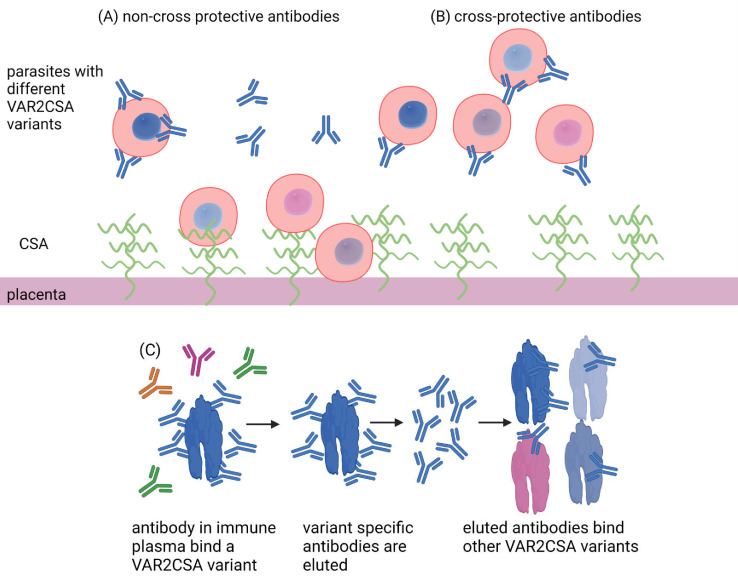
Antibody defence in placental malaria. Placental malaria is caused by an accumulation of parasite-infected red blood cells (red, circled structures) in the placenta. These infected blood cells have a protein (VAR2CSA) that can attach to the CSA protein (green wavy lines) located on the epithelial layer of the placenta (dark pink) (**A**). Over successive pregnancies, the body develops antibodies (Y-shaped proteins) against VAR2CSA that can prevent the infected red blood cells from binding. However, the genetic code of VAR2CSA proteins can vary (illustrated as differently coloured parasites in the red blood cells), and effective antibodies would need to recognize multiple variants. Doritchamou et al. mixed placental plasma from immune mothers with individual VAR2CSA variants and quantified the number of antibodies bound to each one (blue). They then showed that antibodies bound to one full-length VAR2CSA variant were also able to recognize other VAR2CSA variants (**C**) and block their binding to CSA. These antibodies were cross protective (**B**), suggesting that exposure to just a small number of VAR2CSA proteins may be enough to provide protection from placental malaria.

Additionally, naturally occurring antibodies, which lack an attached sugar called fucose, may be more protective than antibodies induced by a placental malaria vaccine, which have this sugar (Larsen et al., 2021). The reasons for this difference are unclear. Perhaps vaccine formulations that better mimic natural antigen presentation, such as providing the full-length protein, would lead to highly active antibodies without fucose, which would be highly desirable.

There is also the question of when is the best time to administer a vaccine. Ideally, immunisation would start in young, adolescent girls, a strategy commonly used for HPV vaccines to prevent cervical cancer. Such a vaccine roll-out would require detailed and ongoing consultation in communities where the vaccine might be used, but saving the lives of young mothers and their babies should be worth every effort.
